# Cell fate decisions of human iPSC-derived bipotential hepatoblasts depend on cell density

**DOI:** 10.1371/journal.pone.0200416

**Published:** 2018-07-10

**Authors:** Nina Graffmann, Audrey Ncube, Wasco Wruck, James Adjaye

**Affiliations:** Institute for Stem Cell Research and Regenerative Medicine, Medical faculty, Heinrich-Heine University, Düsseldorf, Germany; Montana State University Bozeman, UNITED STATES

## Abstract

During embryonic development bipotential hepatoblasts differentiate into hepatocytes and cholangiocytes- the two main cell types within the liver. Cell fate decision depends on elaborate interactions between distinct signalling pathways, namely Notch, WNT, TGFβ, and Hedgehog. Several *in vitro* protocols have been established to differentiate human pluripotent stem cells into either hepatocyte or cholangiocyte like cells (HLC/CLC) to enable disease modelling or drug screening. During HLC differentiation we observed the occurrence of epithelial cells with a phenotype divergent from the typical hepatic polygonal shape- we refer to these as endoderm derived epithelial cells (EDECs). These cells do not express the mature hepatocyte marker ALB or the progenitor marker AFP. However they express the cholangiocyte markers SOX9, OPN, CFTR as well as HNF4α, CK18 and CK19. Interestingly, they express both E Cadherin and Vimentin, two markers that are mutually exclusive, except for cancer cells. EDECs grow spontaneously under low density cell culture conditions and their occurrence was unaffected by interfering with the above mentioned signalling pathways.

## Introduction

*In vitro* differentiation of human pluripotent stem cells (hPSCs) into hepatocyte like cells (HLCs) or cholangiocyte like cells (CLCs) provide valuable tools for modelling hepatogenesis, studying liver-associated diseases, assessing toxicology and for drug screenings. Several protocols have been established to obtain one or the other cell type [1–10]. The success of differentiation highly depends on the quality of the pluripotent stem cells, the initial seeding density of the culture and the proliferation rate of the cells. The ultimate goal is to obtain a pure population of HLCs which have Cytochrome P450 enzyme activity and recapitulate disease associated phenotypes [4–6] or CLCs which are able to form ductual structures in a 3D culture system [7–10].

Bipotential hepatoblasts give rise to hepatocytes and cholangiocytes *in vivo* [11–13]. Hepatocytes are the most abundant cell type in the liver and responsible for metabolism, nutrient storage and drug detoxification. Cholangiocytes are epithelial cells which line the bile ducts that draw through the liver parenchyme and transport bile into the gall bladder. Several signalling pathways have been shown to be involved in the cell fate decision making between hepatocytes and cholangiocytes.

Notch signalling is crucial for the development of cholangiocytes. Impaired Notch signalling due to *JAGGED1* (*JAG1*) or *NOTCH2* mutations causes Alagille Syndrome, a disease that manifests in the liver by a reduction of bile ducts in combination with cholestasis [14–16]. Bile ducts form during liver development next to the portal vein. Bipotential hepatoblasts are specified towards the cholangiocyte fate by Notch signalling, mediated by Notch2 [17, 18]. They form the ductal plate which is the starting point for bile-duct tubulogenesis [17]. Notch signalling in cells adjacent to this first layer of cholangiocytes induces tubulogenesis. After the first ductal structures have formed, all cells lining the duct differentiate towards cholangiocytes [17]. Interestingly, NOTCH3 is the only family member that directs hepatoblasts towards hepatocytes [19].

Susceptibility to Notch signalling depends on transforming growth factor (TGF) β-signalling. As cells of the periportal mesenchyme are major sources for TGFβ secretion, a gradient with decreasing concentrations forms along the periportal-parenchyme axis [20, 21]. Cells near the periportal region are most strongly stimulated by TGFβ and they are the first to form the ductal plate as described above.

Additionally, wingless-type MMTV integration site family (WNT) signalling has been proposed to be involved in hepatic cell fate specification, however, to date contradictory results preclude an unambiguous assignment of its exact role in this process. Several studies have indicated that WNT-β-catenin signalling promotes cholangiocyte and not hepatocyte fate [22, 23], while Cordi *et al*. recently demonstrated that β-catenin is not necessary for biliary development but that its overexpression perturbs cholangiocyte differentiation as well as bile duct morphogenesis [24].

Finally, Hedgehog (Hh) is involved in the complex signalling orchestra that regulates hepatic cell fate. Bipotential hepatoblasts produce and respond to Hh ligands. This dual capacity is retained in cholangiocytes, while healthy hepatocytes lose the ability to produce Hh ligands or to react to its signals. However, upon liver injury they regain the ability to produce Hh ligands [25].

During HLC differentiation *in vitro*, we often observed cells with an epithelial but non-polygonal morphology lacking HLC characteristics occurring at areas of the dish where cell density is low. Here we set out to characterize these cells and applied distinct pathway inhibitors with the aim to reduce their appearance during HLC differentiation and maybe increase the homogeneity of HLC populations.

## Materials and methods

### Ethics statement

The use of iPSC lines for this study was approved by the ethics committee of the medical faculty of Heinrich-Heine University under the number 5013.

### Cell culture

The human ESC line H1 was purchased from WiCell Research Institute (Madison, WI, USA), human iPSCs were generated as described in [26, 27].

**Fig 1:**
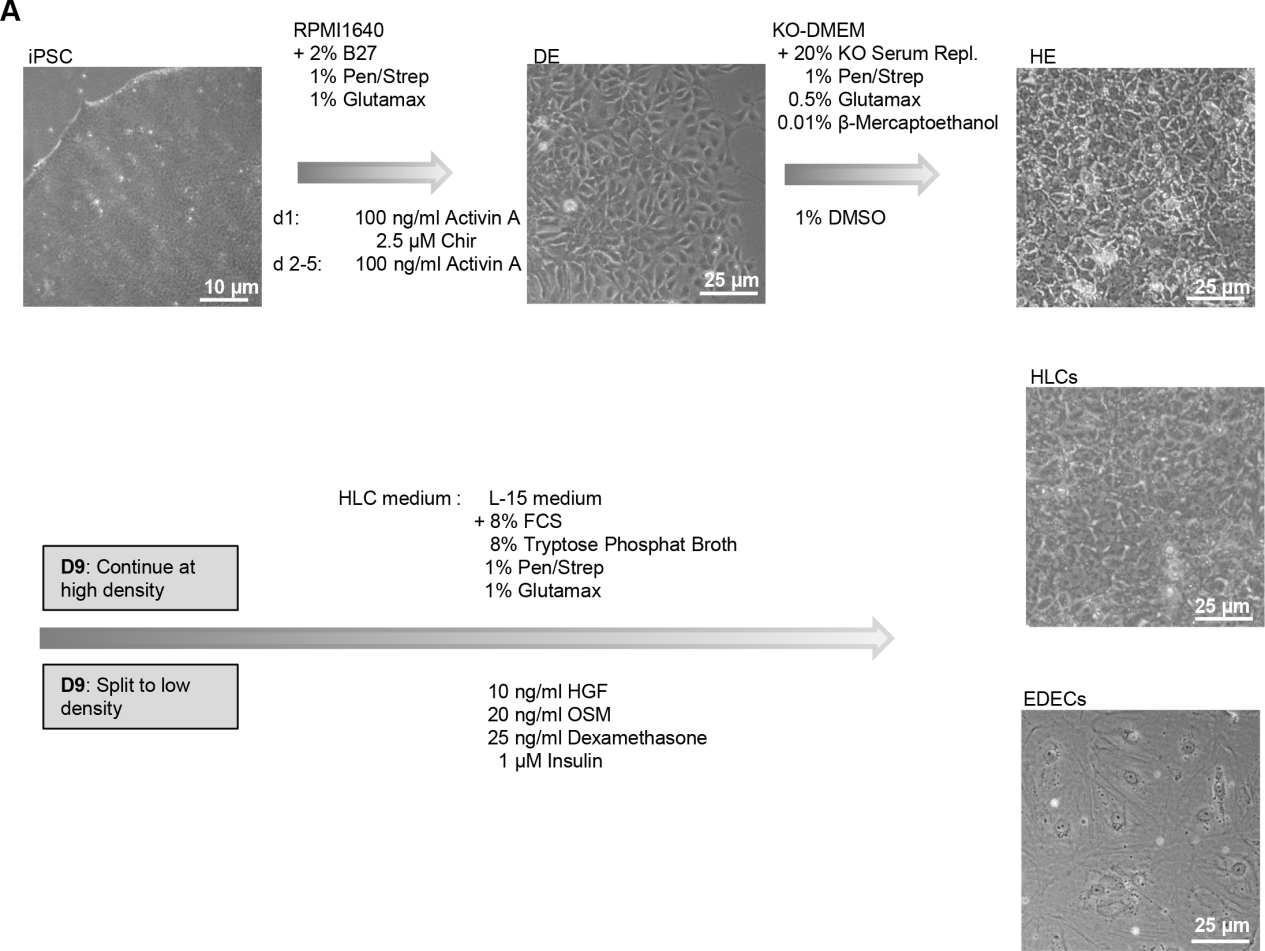
Differentiation of hPSCs into hepatocyte like cells (HLCs) and endoderm derived epithelial cells (EDECs). hPSCs were differentiated into hepatic endoderm (HE) which consists of bipotential hepatoblasts. Afterwards, cultures were either continued unperturbed in order to obtain HLCs, or split and replated at low density to obtain EDECs. Morphological changes were documented for each stage.

hPSCs were cultured on matrigel (Corning) coated plates with Stem MACS (Miltenyi) or TSR E8 medium (Stemcell Technologies). Medium was changed on a daily basis. Spontaneously differentiated cells were removed manually if necessary. For differentiation, cells were split onto matrigel coated plates and kept in the stem cell medium for another 16-24h. Afterwards, HLC differentiation was performed as described previously [1]. In brief, cells were first differentiated towards definitive endoderm (DE) using DE medium: 96% RPMI 1640, 2% B27 (without retinoic acid), 1% Glutamax (Glx), 1% Penicillin/Streptomycin (P/S) (all Gibco), 100 ng/ml Activin A (Peprotech) and for the first day 2.5 μM Chir99021 (Tocris). After 5 days the medium was changed to one favouring hepatic endoderm (HE): 78% Knockout DMEM, 20% Knockout serum replacement, 0.5% Glx, 1% P/S, 0.01% 2-Mercaptoethanol (all Gibco) and 1% DMSO (Sigma), which was used for an additional 4 days. In order to induce endoderm derived epithelial cells (EDEC) differentiation, HE cells were split and plated onto matrigel coated plates on day 9 of the differentiation. Cell density has to be low to induce EDEC differentiation, which was achieved by seeding 25,000/cm^2^ after splitting at the HE stage. On day 10 the differentiation was continued with HLC medium: 82% Leibovitz 15 medium, 8% fetal calf serum, 8% Tryptose Phosphate Broth, 1% Glx, 1% P/S (all Gibco) with 1 μM Insulin (Sigma), 10 ng/ml hepatocyte growth factor (HGF) (Peprotech), 20 ng/ml Oncostatin M (OSM) 209 a.a. (Immunotools), 25 ng/ml Dexamethasone (DEX) (Sigma) (Fig 1A). During the course of differentiation towards the DE, HE and the beginning of the HLC/EDEC stage, medium was changed daily, but at later time points every other day.

In order to switch cell fate after HE stage, several signaling pathways were inhibited or activated with small molecules listed in S1 Table.

### Immunocytochemistry

Cells were fixed with 4% paraformaldehyde for 15 min. Unspecific binding sites were blocked by incubating 2 h at room temperature with blocking buffer (1x PBS with 10% normal goat or donkey serum, 1% BSA, 0.5% Triton and 0.05% Tween). Antibodies were diluted in blocking buffer diluted 1:2 with 1x PBS (S2 Table). Primary antibodies incubated overnight at 4°C. Cells were washed three times with 1x PBS/ 0.05% Tween and incubated with the secondary antibody for 2 h at room temperature. Cells were washed as above and images captured using a fluorescence microscope (LSM700, Zeiss). For extracellular stainings blocking and wash buffer without detergents were used. DNA was stained with Hoechst 33258 (Sigma). Individual channel images were processed and merged with Photoshop CS6 or Fiji.

#### RNA isolation and quantitative real time PCR (qRT-PCR)

Up to 500,000 cells were lysed in 500 μl Trizol and RNA was isolated with the Direct-zol™ RNA Isolation Kit (Zymo Research) according to the user’s manual. On-column DNase digestion was performed. 500 ng of RNA were transcribed into cDNA using the TaqMan Reverse Transcription (RT) Kit (Applied Biosystems). In the case of H1 derived EDECs also cRNA obtained from Affymetrics Array preparation was transcribed into cDNA using the TaqMan RT kit with random hexamers instead of oligo-dT primers. Primers for qRT-PCR were purchased from MWG (S3 Table). Real time PCR was performed in technical triplicates of biological duplicates with Power Sybr Green Master Mix (life technologies) on a VIIA7 (life technologies). In the case of H1 derived EDECs with and without inhibitor only one biological sample was analyzed. Mean Ct values were normalized to RPS16 as a housekeeping gene and fold change was calculated relative to the controls. Results are depicted as mean values (log2) with standard error of the mean (SEM). P-Values were calculated with two-tailed student’s t-tests (*** = p-value < 0.001, ** = p-value < 0.01, * = p-value < 0.05).

### Transcriptome and bioinformatics analysis

Microarray experiments were performed employing the Affymetrix PrimeView chip (BMFZ, Düsseldorf). Details of data analysis are given in Supplementary materials and methods.

### Biochemical activity assays

Cytochrome P450 3A4 (CYP3A4) activity was measured in technical triplicates with the respective P450 Glo assay from Promega, according to the recommendations. Supernatants were stored from every step of the differentiation process and urea content was measured in technical duplicates of biological duplicates with the QuantiChrom Urea Assay (Bioassay systems) according to the manufacturer’s recommendations. Results are depicted as mean values with standard error of the mean (SEM) in case of the CYP3A4 assay and standard deviation in case of urea measurement. P-Values were calculated with two-tailed student’s t-tests (*** = p-value < 0.001, ** = p-value < 0.01, * = p-value < 0.05).

## Results and discussion

### Differentiation of hPSCs in high and low density conditions

During *in vitro* differentiation of hPSCs into hepatocyte like cells (HLCs), we frequently observed cells with atypical morphology, predominantly at the borders of densely grown colonies. As these cells only occur at regions of low cellular density, we reasoned that they require only lose cell-cell contact in combination with enough space for growth. The cells are of endodermal origin and have an epithelial morphology but are much larger than HLCs or cholangiocyte like cells (CLCs). In order to characterize these endoderm derived epithelial cells (EDECs), we tried to produce a pure population by reducing cellular density during HLC differentiation. To this end, we differentiated two iPSC lines as well as H1 ESCs into bipotential hepatoblasts by following our recently published protocol for hepatocyte differentiation until the stage of hepatic endoderm (HE) [1]. The iPSC lines were derived from human fetal foreskin (HFF) [26] and human amniotic fluid (AF) cells [28], respectively. hPSCs changed their morphology upon induction of definitive endoderm (DE) and the typical loose and petal-like morphology became visible (Fig 1A upper row, middle panel). HE induction started after five days. At the end of this stage, the morphology of the cells resembled the typical polygonal structure of hepatocytes (Fig 1A upper row, right panel). We then either split and replated the HE cells at a low density in order to obtain an enriched population of the uncharacterized cells or we left the densely populated plates untouched for obtaining HLCs. From the next day on, culture medium was replaced with HLC medium and differentiation was continued for an additional five days. Cells cultivated at a high density only marginally changed their morphology and maintained the polygonal morphology which is typical of HLCs (Fig 1A middle row, right). However, cells split and replated at low density underwent dramatic morphological changes. They still had the typical epithelial cell-cell contact but were rather large with a flat and irregular shape. Interestingly, pronounced intracellular structures, which resemble parts of the cytoskeleton, as well as dark granula became visible (Fig 1A lowest row, right).

### EDECs resemble CLCs but are Vimentin positive

**Fig 2:**
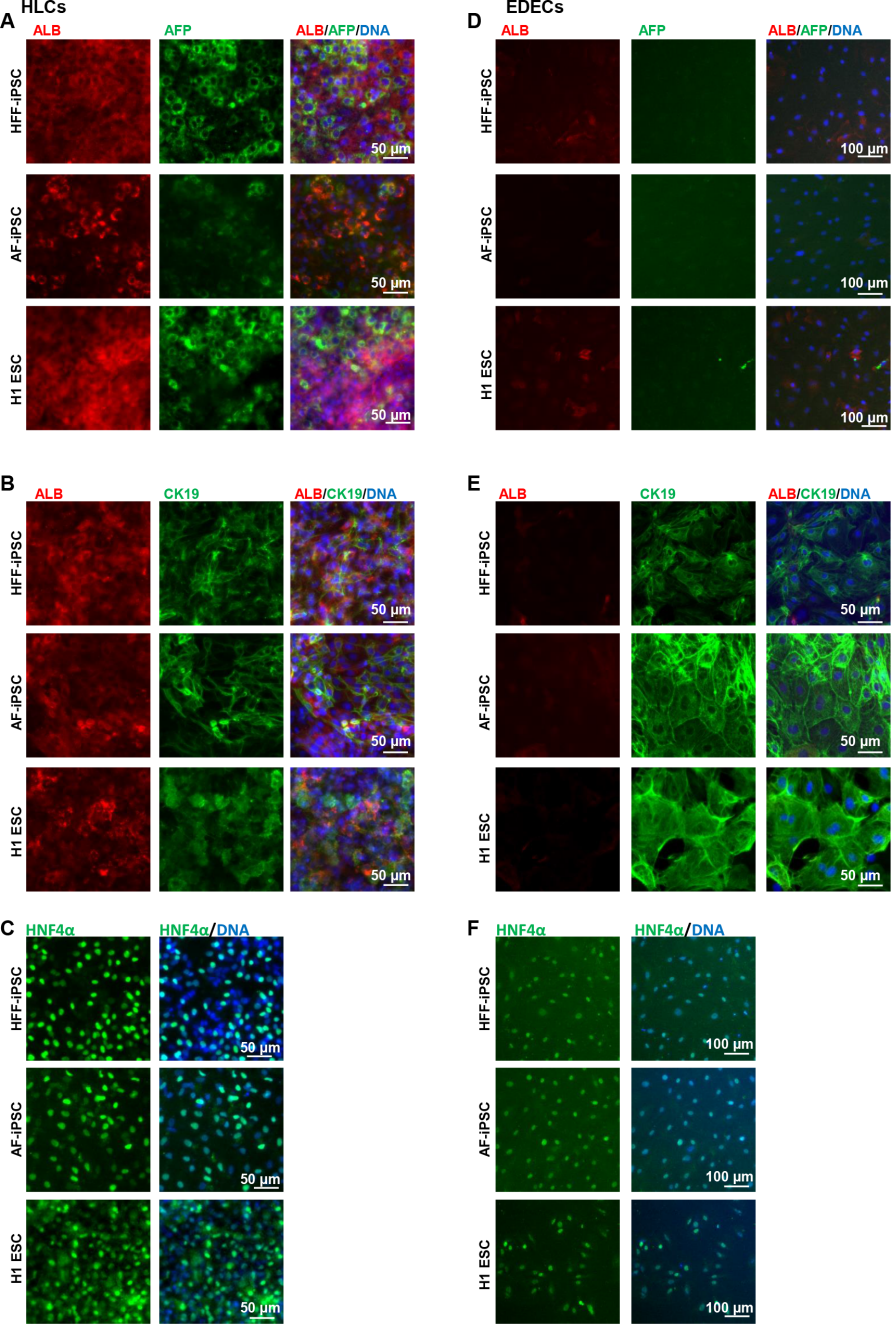
Expression of characteristic hepatocyte markers in HLCs and EDECs. Two iPSC lines and one ESC line were differentiated into either HLCs (A-C) or EDECs (D-F) and stained for the expression of characteristic hepatocyte markers.

The two iPSC lines as well as H1 ESCs were differentiated into HLCs and EDECs and stained for expression of characteristic markers at the respective end-stages. HLCs expressed Albumin (ALB) and Alpha Fetoprotein (AFP) as well as Cytokeratin 19 (CK19) and HNF4α (Fig 2A–2C), while EDECs were negative for ALB and AFP but expressed high levels of CK19 as well as HNF4α (Fig 2D–2F).

**Fig 3:**
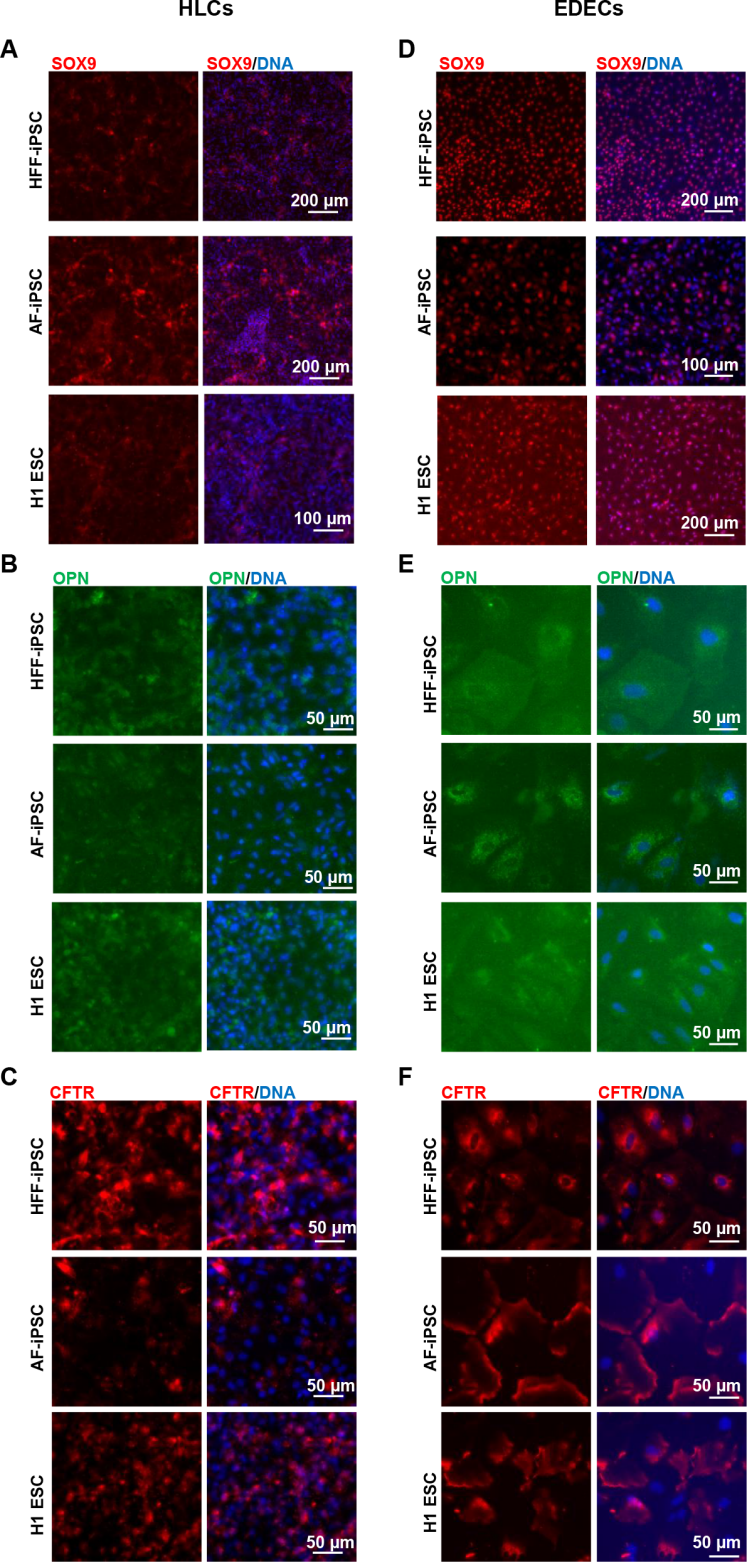
Expression of characteristic cholangiocyte markers in HLCs and EDECs. Two iPSC lines and one ESC line were differentiated into either HLCs (A-C) or EDECs (D-F) and stained for the expression of characteristic cholangiocyte markers.

To test whether EDECs are related to cholangiocytes, we analysed the expression of characteristic markers for this cell type by immunocytochemistry. The cholangiocyte specific transcription factor SOX9 and the multifunctional protein osteopontin (OPN) were almost not present in HLCs at the protein level (Fig 3A and 3B). However, we could detect low level expression of the transporter Cystic Fibrosis Transmembrane Conductance Regulator (CFTR), but the protein was not as expected localized within the membrane, but in the cytoplasm (Fig 3C).

In contrast, EDECs expressed SOX9 as well as weak levels of OPN and they were highly positive for CFTR which is clearly localized within the cell membrane as expected in cholangiocytes (Fig 3D–3F).

**Fig 4:**
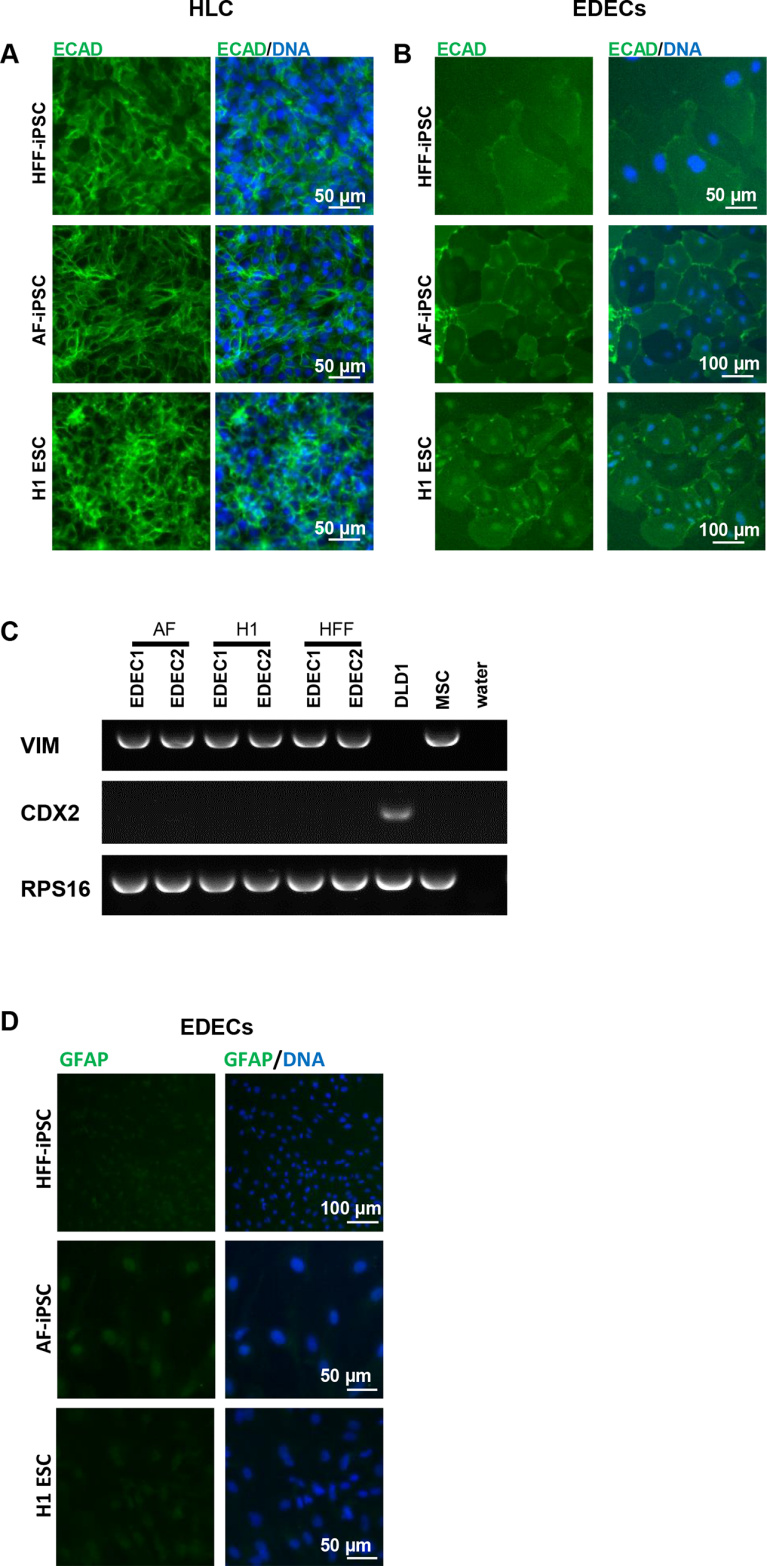
EDECs express a unique combination of markers. Two iPSC lines and one ESC line were differentiated into either HLCs (A) or EDECs (B-D) and marker expression was analysed. (A,B) Immunocytochemistry for ECAD. (C) Endpoint RT-PCR for *VIM* and *CDX2*. cDNA derived from mesenchymal stem cells (MSCs) and from the colon cancer line DLD1 served as positive controls for *VIM* and *CDX2* expression, respectively. (D) Immunocytochemistry for GFAP.

Both HLCs and EDECs expressed E-Cadherin (ECAD), which is characteristic of epithelial cells (Fig 4A and 4B). However, RT-PCR revealed that they also expressed *Vimentin* (*VIM*, Fig 4C) an intermediate filament protein which defines mesenchymal cells. Its co-expression with ECAD has so far only been described in cancerous cells which undergo epithelial-to-mesenchymal transition [29]. EDECs were negative for *CDX2* expression, a transcription factor that is characteristic of intestinal cells (Fig 4C). GFAP, a marker for stellate cells, was only marginally expressed (Fig 4D).

HE cells which have the potential to differentiate into HLCs, CLCs and also EDECs were positive for all investigated markers that are known to be expressed in the immature stages of either HLCs or EDECs, namely AFP, CK19, HNF4α, SOX9, and ECAD (S1A–S1E Fig). However, more mature markers such as ALB, CFTR, and OPN were either inconsistently expressed or mislocalized (S1A, S1C and S1F Fig). In addition, HE cells were negative for GFAP (S1G Fig).

**Fig 5:**
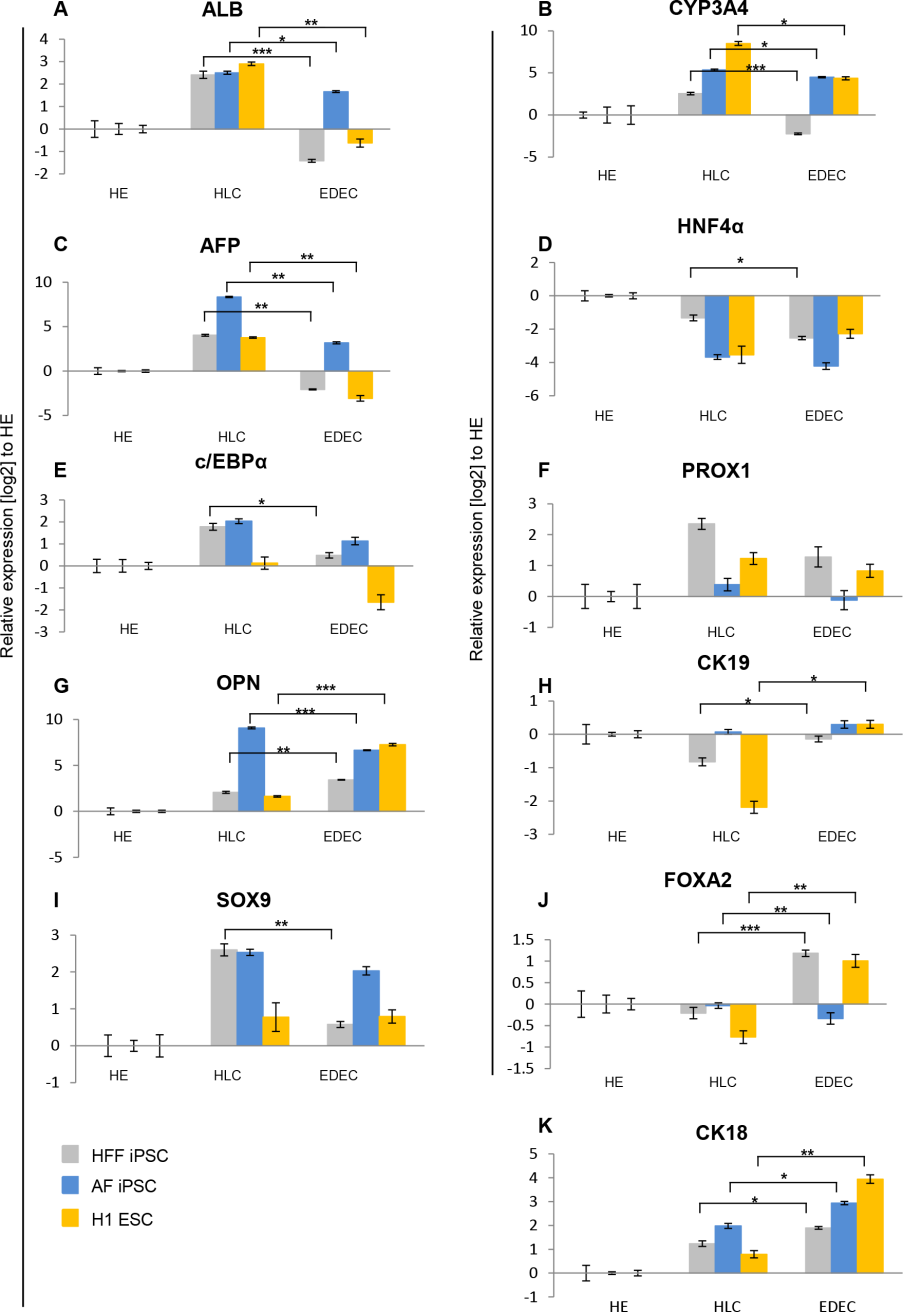
Temporal expression of markers during differentiation. Two iPSC lines and one ESC line were differentiated into either HLCs or EDECs and qRT-PCRs for expression of characteristic hepatocyte and cholangiocyte markers was performed. Gene expression was normalized to RPS16 and fold change was calculated relative to HE cells. Mean values of technical triplicates of biological duplicates are show. Error bars represent SEM. P-Values were calculated with two-tailed student’s t-tests (*** = p-value < 0.001, ** = p-value < 0.01, * = p-value < 0.05).

qRT-PCR revealed that crucial mature (*ALB*, *CYP3A4*) as well as immature (*AFP*) hepatocyte markers were significantly up-regulated in HLCs compared to EDECs and HE cells (Fig 5A–5C). Expression of *HNF4α*, which marks early hepatic differentiation stages and mature hepatocytes, was highest in HE cells. This might imply that these cells are poised to differentiate along the hepatic lineage (Fig 5D). Compared to HE cells, EDECs expressed lower levels of *HNF4α* (Fig 5D). Both *c/EBPα* and *PROX1*, which are transcription factors that promote HLC fate over EDEC were expressed at higher levels in HLCs than in EDECs, although in almost all cases not significantly (Fig 5E and 5F). The classic cholangiocyte marker OPN was, with the exception of AF iPSC derived cells, significantly expressed at higher levels in EDECs than in HLCs, as was the cholangiocyte-associated intermediate filament protein -CK19 (Fig 5G and 5H). Unexpectedly, EDECs expressed lower levels of the cholangiocyte specific transcription factor *SOX9* than HLCs (Fig 5I).

The transcription factor FOXA2 which promotes hepatocyte fate and limits cholangiocyte proliferation was also expressed higher in most of the EDECs than in HLCs which does not support a similarity with cholangiocytes (Fig 5J). EDECs also express higher levels of CK18 than HLCs, even though this intermediate filament protein is enriched in hepatocytes (Fig 5K).

We measured cytochrome P450 (CYP3A4) activity as well as urea synthesis in order to check if EDECs might have hepatocyte-associated functions. HFF- and H1 derived HLCs had the highest levels of CYP3A4 activity (S2A Fig). In both cases, HE cells showed significantly lower activity and in EDECs only minimal activity was measurable. In the case of AF-derived cells, CYP3A4 activity was in all three stages non-significant (S2A Fig). EDECs derived from HFF-iPSCs did not produce any urea, while HLCs were highly active (S2B Fig). Overall, these data indicate that low density conditions during HLC differentiation result in cells which are morphologically and functionally clearly not hepatocytes, although they express some hepatocyte-associated markers. On the other hand they express several cholangiocyte markers in combination with Vimentin. Taken together, the pattern of marker expression implies that EDECs are an immature cell type with intermediate characteristics between HLCs and CLCs.

Interestingly, similar cell types are present in rat fetal liver where three distinct populations, comprising cells either expressing AFP, ALB and CK19, or AFP and ALB or only CK19 have been associated with distinct lineage commitment and re-population capacities [30].

### Inhibition of Notch signalling does not impair EDEC formation but alters expression of key genes

**Fig 6:**
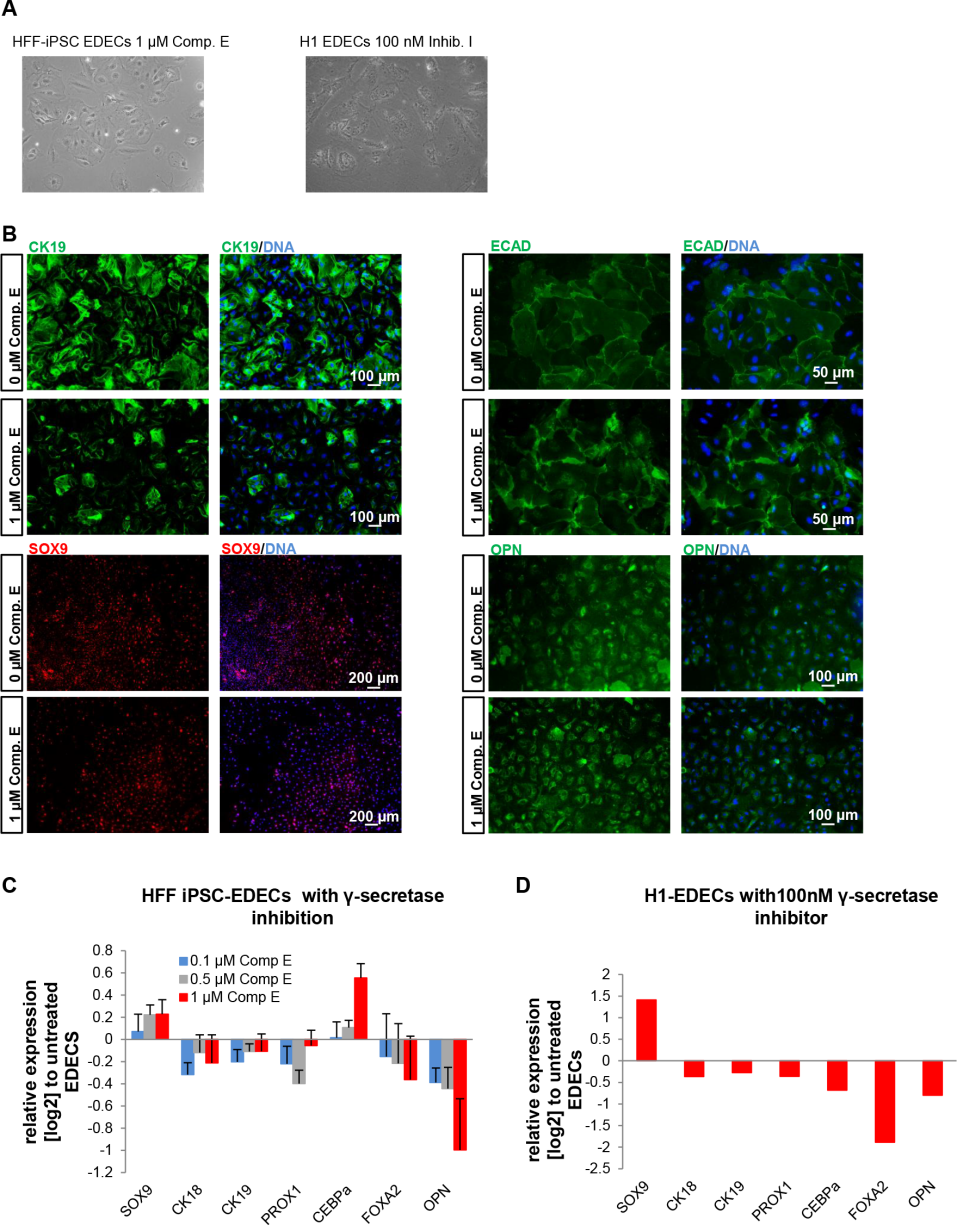
Inhibition of Notch signalling does not prevent EDEC development. hPSCs were differentiated into EDECs while treating them with γ-secretase inhibitors. (A) HFF derived cells treated with Compound E (left) or H1 derived cells treated with γ-secretase inhibitor I (right) adopted the EDEC morphology. (B) Immunocytochemistry for CK19, SOX9, ECAD, and OPN. (C,D) qRT-PCR for hepatocyte or cholangiocyte determining genes. Gene expression was normalized to RPS16 and fold change was calculated relative to untreated cells. Mean values of technical triplicates of biological duplicates (C) or of one sample (D) are show.

Notch signalling has been described during liver development as the most important signalling pathway for the determination of cholangiocyte fate in contrast to hepatocyte [17, 18]. Therefore, we assumed that blocking Notch signalling might push cells into HLC direction even at a low density. An essential and universal step in Notch signalling is cleavage of the membrane bound Notch receptor by γ-secretase after ligand binding [31]. This creates the Notch intracellular domain which activates the transcription of specific target genes [31]. In order to prevent EDEC differentiation, we blocked Notch signalling by inhibiting γ-secretase activity. After splitting the cells and replating at low density we incubated them with two distinct γ-secretase inhibitors and continued differentiation for an additional six days. HFF-iPSC derived hepatoblasts were treated with Compound E while H1 derived hepatoblasts were incubated with γ-secretase inhibitor I. At the end of the differentiation, cells cultivated at low density predominantly adopted an EDEC phenotype, regardless of Notch inhibition, even though cells with typical HLC morphology were also visible (Fig 6A). Immunostainings confirmed the predominance of EDECs, as most cells continued to express high levels of CK19 and SOX9 as well as low levels of ECAD and OPN (Fig 6B).

We also looked in detail at the expression of several HLC and CLC specific/ enriched genes after treatment with γ-secretase inhibitors. With the exception of *c/EBPα* we observed similar regulation for all genes analysed in both cell lines, but because of the limited number of samples analysed, these expression changes are not significant (Fig 6C and 6D). Interestingly, both γ-secretase inhibitors had opposing effects on the two most characteristic cholangiocyte markers- *SOX9* and *OPN*. *SOX9* was up-regulated while *OPN* was down-regulated. Treatment with different concentrations of Compound E revealed that both effects were dose dependent (Fig 6C). SOX9 is one of the earliest markers for cholangiocyte specification [32] and might thus not be entirely dependent on Notch signalling. OPN, however, is a more mature marker which is expressed later than SOX9 [32]. It was overall only weakly expressed in our cells and its down-regulation upon γ-secretase inhibition might imply impaired maturation.

We further analysed three other transcription factors involved in hepatic cell fate determination in more detail. *PROX1* and *FOXA2* were uniformly down-regulated in iPSC and H1 derived EDECs after γ-secretase inhibition. The homeobox transcription factor PROX1 promotes hepatocyte fate and represses biliary fate [33]. FOXA2, which is important for hepatocyte development is known to repress cholangiocyte proliferation [34]. The down-regulation of both factors after γ-secretase inhibition supports the immunocytochemistry-based data which shows that the cells do retain EDEC fate. Expression of *c/EBPα* changed in opposing directions after γ-secretase treatment of iPSC and H1-derived EDECs. It is known that Notch signalling down-regulates c/EBPα expression in cholangiocytes [35]. Thus, the dose-dependent up-regulation that we observed in iPSC- derived EDECs after treatment with Compound E would imply that Notch signalling was effectively reduced. Maybe the concentration of γ-secretase inhibitior I was not high enough to achieve similar results with the H1 derived EDECs. The transcription factor c/EBPα has a versatile role during development of hepatocytes and cholangiocytes. It activates on the one hand transcription of characteristic hepatocyte markers such as albumin and several enzymes of the ornithine cycle [36], while limiting hepatocyte proliferation [37] and it inhibits cholangiocyte fate by suppressing expression of the cholangiocyte determining transcription factors- HNF6 and HNF1β [38]. Thus, effects of different c/EBPα levels are probably not visible at this early, immature stage of our cells.

**Fig 7:**
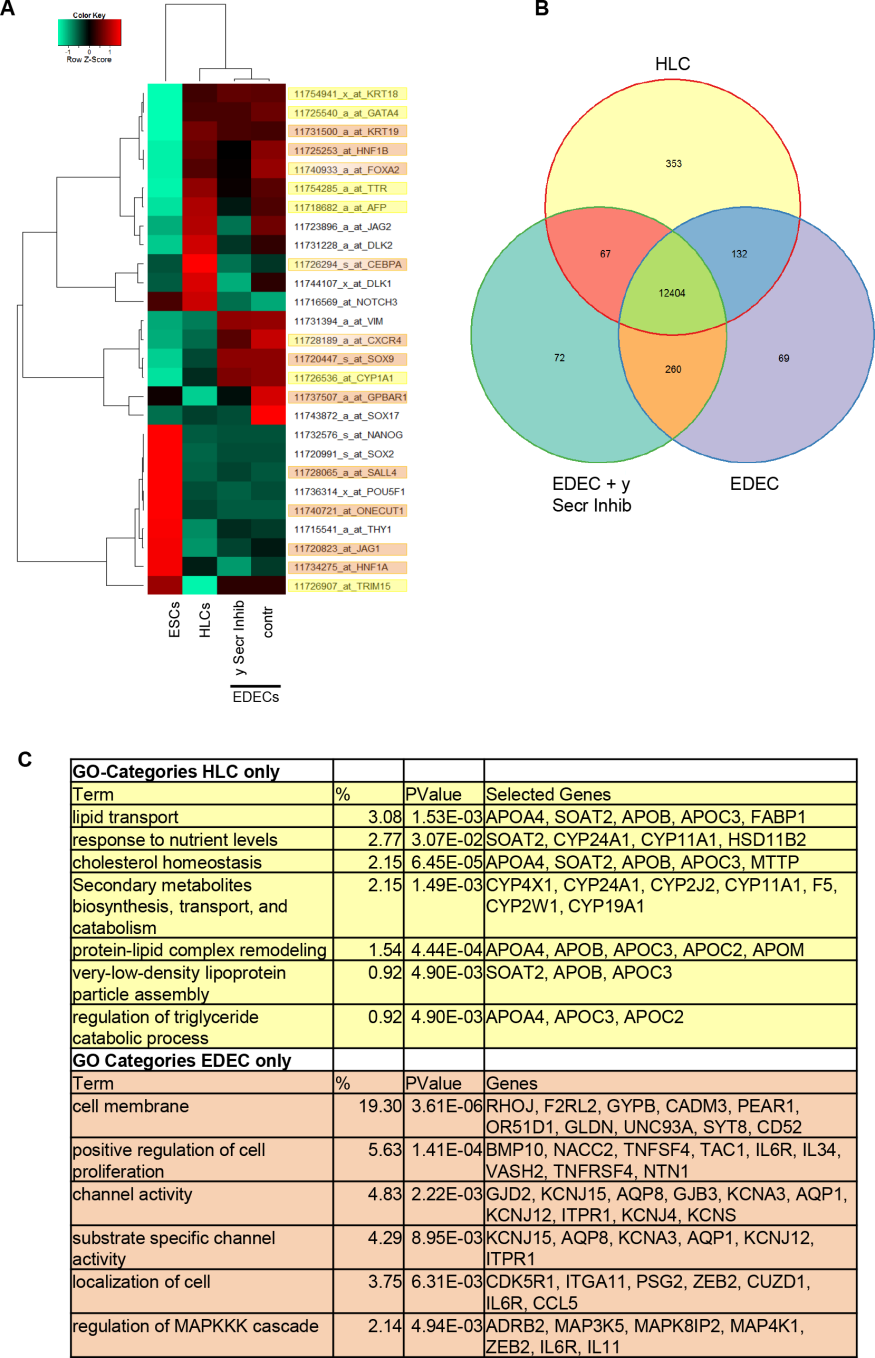
Gene expression analysis unravels differences and similarities between H1 derived HLCs and EDECs. (A) Heatmap representation of key genes involved in hepatic cell fate decision making or characteristic for either hepatocytes (yellow) or cholangiocytes (orange). (B) Venn diagram illustrating the numbers of genes which were expressed either by only one cell type or shared between the cell types. (C) Gene Ontology (GO) analysis of genes expressed only in HLCs (yellow) or in EDECs (with or without Notch inhibition, orange) Shown are pre-selected, significant GO-Terms, for full data set see S4 and S5 Tables.

Microarray-based global gene expression analysis revealed that the transcriptomes of H1 derived HLCs cluster away from H1 derived EDECs regardless of Notch inhibition in the latter (Fig 7A). This further emphasizes the diversity of the two observed cell types. On the other hand, heatmap-based analysis of hepatocyte- and cholangiocyte- associated genes revealed a close relationship between HLCs and EDECs (Fig 7A). Many of the analysed transcription factors were expressed at similar levels in both cell populations. Interestingly, *c/EBPα* was clearly over-expressed in HLCs while *GPBAR1* and *SOX17* expression were higher in EDECs. c/EBPα is a transcription factor that inhibits cholangiocyte fate by suppressing expression of the cholangiocyte determining transcription factors HNF6 and HNF1β [38]. G Protein-Coupled Bile Acid Receptor 1 (GPBAR1 also known as TGR5) has been shown to be characteristic for cholangiocytes [39], while the presence of SOX17, an early marker for endoderm, indicates that the cells are still immature.

Surprisingly, members of the Notch signalling pathway (*NOTCH3*, *JAG2* and *DLK1*,*2*) were expressed at higher levels in HLCs than in EDECs. This was unexpected as it has been described that hepatocyte differentiation does not require Notch signalling. HLCs are however still immature and the presence of molecules belonging to the Notch signalling pathway might indicate that they are still able to switch cell fate towards CLCs or EDECs. In particular, the presence of DLK1 has been described as being characteristic for bipotential hepatoblasts [40]. NOTCH3 is the only analysed factor of this signalling pathway that is up-regulated in EDECs after treatment with the γ-secretase inhibitor. As inhibition of the γ-secretase inhibits propagation of the Notch signal cells might reduce the production of ligands which do not find a receptor counterpart, while the NOTCH3 receptor might be up-regulated in order to compensate for low ligand density on neighbouring cells. Interestingly, NOTCH3 is also the only family member with a positive impact on hepatocyte development [19] and its increased expression after γ-secretase inhibition might indicate that some of the cells are still or again capable of differentiating into hepatocytes.

Global gene expression was compared between HLCs and EDECs with and without treatment with Notch inhibitor (Fig 7B). While 12,404 genes were expressed by all three cell types, 353 genes were only expressed in HLCs. EDECs and EDECs treated with the Notch inhibitor shared 260 genes and individually expressed 69 and 72 genes, respectively.

Genes only expressed in either HLCs (yellow) or EDECs (comprising both, untreated and treated with γ-secretase inhibitor; orange) were assigned to distinct gene ontology (GO) terms (Fig 7C, S5 Table). Interestingly, in the case of HLCs many of these terms relate to metabolic functions while in EDECs, structural features and signalling pathways are predominant. This again confirms the presence of two distinct cell types in our culture dish. There was a general tendency of EDECs expressing fewer genes related to transcription and cell cycle than HLCs while expressing more genes related to apoptosis and proliferation (S6–S8 Tables).

Analysis of differentially expressed genes between EDECs and EDECs treated with γ-secretase inhibitor I revealed that untreated cells expressed more genes related to development and differentiation in general (S9–S11 Tables). This is in line with Notch inhibition reducing the possibilities of cellular differentiation and interfering with development. In addition, γ-secretase inhibitor I treated EDECs expressed more genes related to cell cycle which mirrors the reduced differentiation/development pathways which would lead cells towards more maturity and thus less cell cycling. Interestingly, inhibition of Notch signalling resulted in a general reduction of genes associated with cell signalling affecting also other signalling pathways (S9–S11 Tables).

### Inhibition of key signalling pathways does not revert cell fate

**Fig 8:**
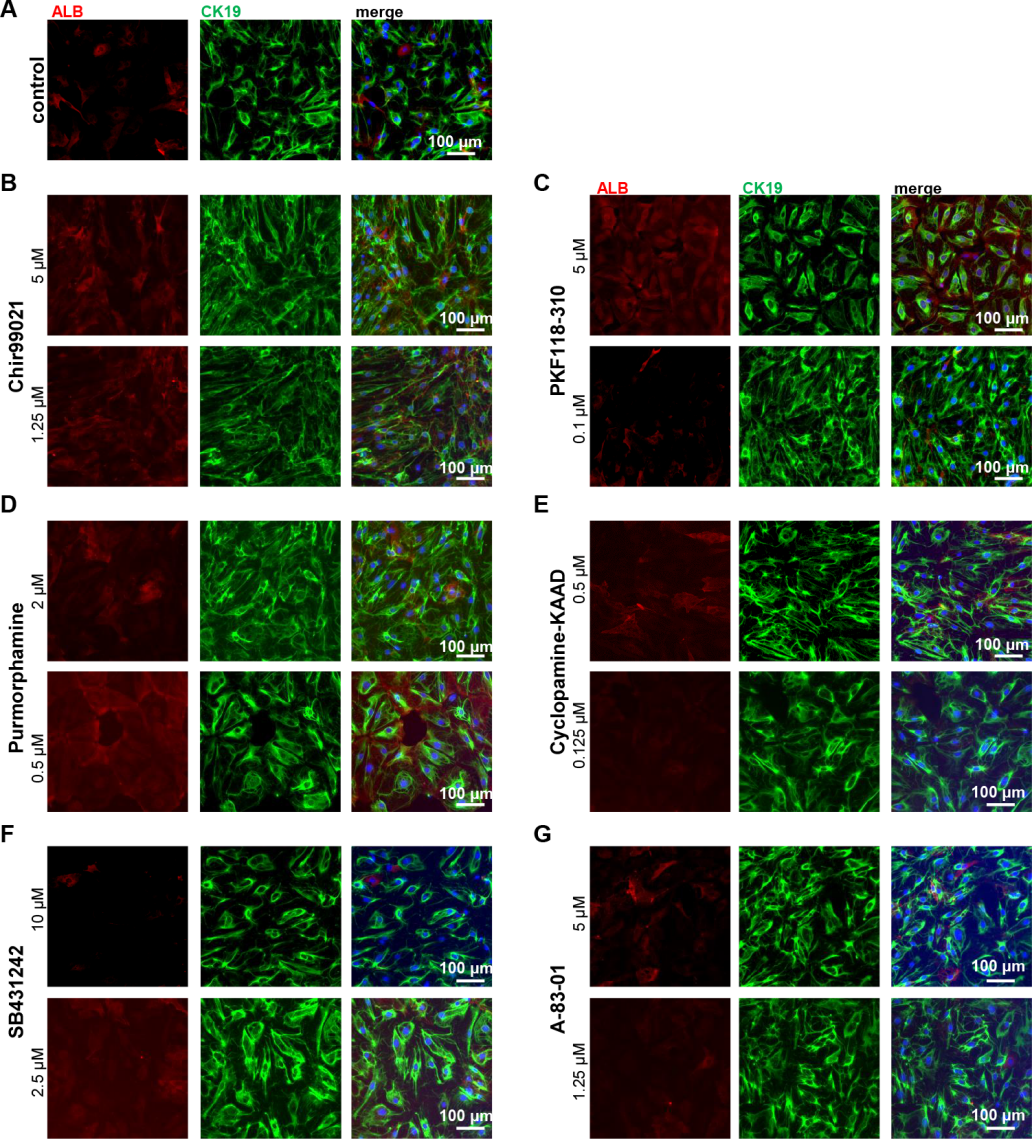
Interference with various signalling pathways does not change cell fate. iPSCs were differentiated into EDECs. Directly after low-density splitting, small molecules were applied in order to interfere with signalling pathways important for differentiation into hepatocytes or cholangiocytes. (A-G) Immunocytochemistry for ALB (red) and CK19 (green). (A) DMSO control, (B) activation of WNT signalling with Chir99021, (C) inhibition of WNT signaling with PKF118-310, (D) activation of Hh signalling with Purmorphamine, (E) inhibition of Hh signalling with Cyclopamine-KAAD, (F) inhibition of TGFβ signalling with SB431242, (G) inhibition of TGFβ signalling with A-83-01. Scale bar: 100 μm.

We next wanted to determine whether interference with one of the other major signalling pathways that have been described as essential for cell fate decision making between hepatocytes and cholangiocytes can revert the EDEC phenotype and push the cells into the HLC direction. To this end, we applied several small molecules to either induce or repress WNT, Hh, and TGFβ signalling (Fig 8A–8G). As with Notch inhibition, we did not observe changes in the phenotype and in all cases the cells were predominantly EDECs. However, the population was not pure, and we could also detect cells with typical HLC morphology. We performed immunocytochemistry-based expression analyses for ALB and CK19 in all conditions, (Fig 8B–8G) and compared it to untreated control cells (Fig 8A). This demonstrated that the majority of cells expressed high levels of CK19 and low levels of ALB. We confirmed in our transcriptome data, that the cells expressed the essential proteins for all investigated signalling pathways thus being able to react to the applied cues (S3 and S4 Fig).

Overall, it seems that splitting and replating pluripotent stem cell derived bipotential hepatoblasts at low density at the HE stage of the HLC differentiation process leads to the accumulation of EDECs. None of the applied pathway interferences was able to revert or prevent this transition. However, cell fate decision is a tightly orchestrated process with a high level of synergy between different signalling pathways. Thus, it is possible that the right interplay between the various pathways at the right time point is capable of altering cell fate. In order to determine the full potential of these cells and classify them according to already known categories of hepatic stem/progenitor cells, transplantation experiments with rats or mice after partial hepatectomy will be necessary.

As cells with EDEC morphology often appear during HLC differentiation in regions of lower cell density, it is essential to keep the cells during the whole differentiation process at a high density. The accumulation of EDECs within a population of HLCs might interfere with disease modelling or drug screenings specifically related to hepatocytes.

## Supporting information

S1 Fig Expression of characteristic markers in HE cells. Two iPSC lines and one ESC line were differentiated into HE cells and stained for characteristic hepatocyte or cholangiocyte markers.
(TIF)

S2 Fig Biochemical activity tests. hPSC were differentiated into HLCs and EDECs. (A) CYP3A4 activity assay (B) QuantiChrome Urea Assay.
(TIF)

S3 Fig Heatmap representation of genes involved in signalling pathways part 1. Global expression patterns of genes involved in Notch (A) and Hedgehog (B) signalling were analysed in HLCs and EDECs with and without Notch inhibitor. Genes were colour-coded according to their function. Asterisks mark the genes that are expressed above threshold in at least the EDEC sample or the EDEC sample with inhibitor.
(TIF)

S4 Fig Heatmap representation of genes involved in signalling pathways part 2. Global expression patterns of genes involved in WNT (A) and TGFβ (B) signalling were analysed in HLCs and EDECs with and without Notch inhibitor. Genes were colour-coded according to their function. Asterisks mark the genes that are expressed above threshold in at least the EDEC sample or the EDEC sample with inhibitor.
(TIF)

S1 Table Small molecules. (DOCX)

S2 Table Antibodies. (DOCX)

S3 Table Primer sequences. (DOCX)

S4 Table Venn sets. The genes included in the different sets of the venn diagram shown in Fig 5D are listed in this table.
(XLS)

S5 Table Common GO terms in H1 HLCs and EDECs. Genes expressed either in HLCs or in EDECS (regardless of inhibitor treatment) from the venn diagram (Fig 5D) were used for GO analysis. Clusters are listed in this table.
(XLSX)

S6 Table Selected GO Categories up- and down regulated in EDECs versus HLCs. (DOCX)

S7 Table Comparison of gene expression between EDECs and HLCs. (XLSX)

S8 Table Comparison of gene expression between EDECs and EDECs treated with γ-secretase inhibitor. (XLSX)

S9 Table Selected GO categories up- and down regulated in EDECs with y-secretase inhibitor versus untreated EDECs. (DOCX)

S10 Table GO Terms of genes expressed in both, EDECs and HLCs. (XLSX)

S11 Table GO Terms of genes expressed in both, EDECs and EDECs treated with γ-secretase inhibitor. (XLSX)
