# Supplementary figures and images for: Cell fate decisions of human iPSC-derived bipotential hepatoblasts depend on cell density
Source: PLoS One. 2018 Jul 10;13(7):e0200416. doi: 10.1371/journal.pone.0200416 (PMC6039024; doi:10.1371/journal.pone.0200416)

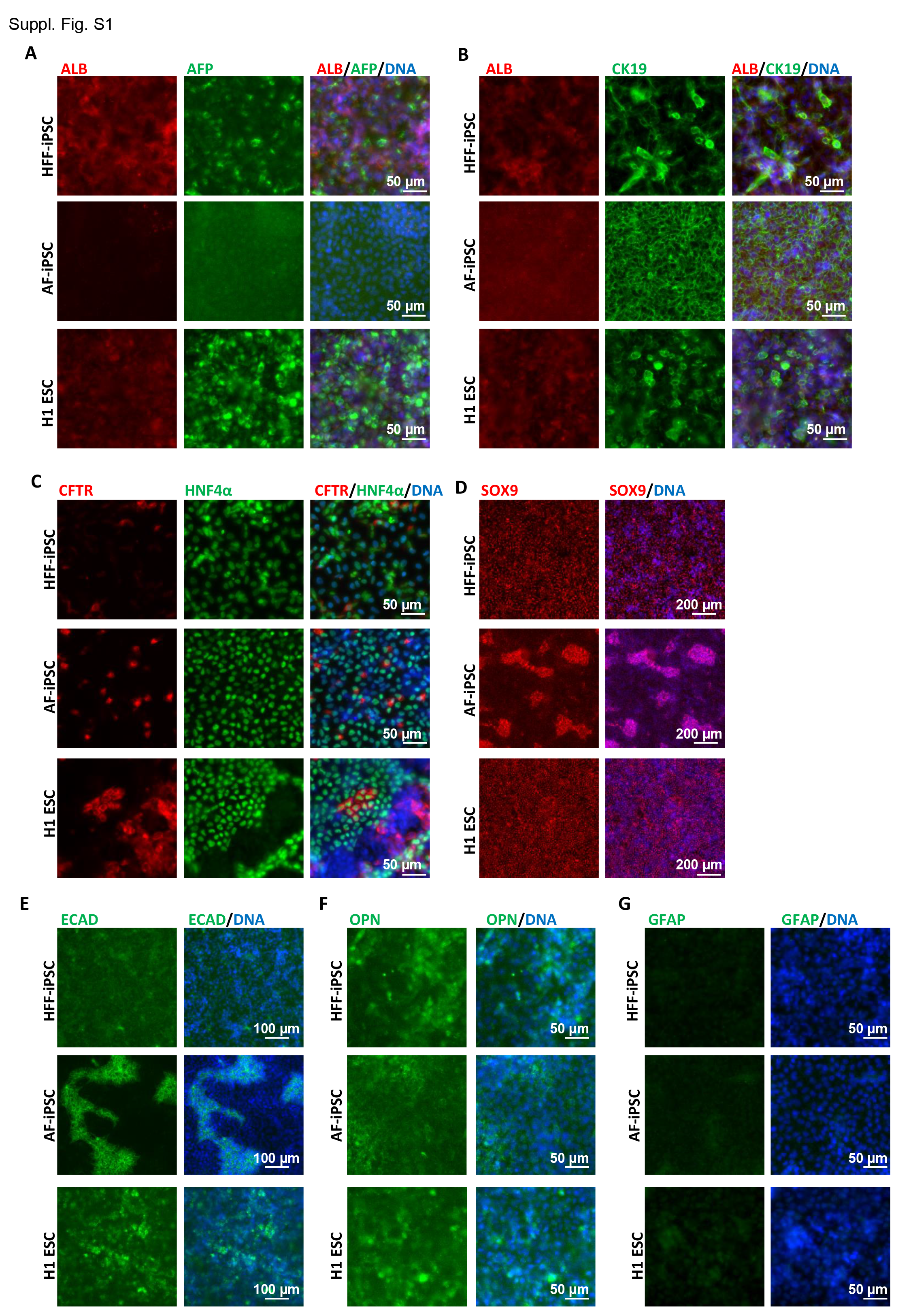

Supplement: S1 Fig — Two iPSC lines and one ESC line were differentiated into HE cells and stained for characteristic hepatocyte or cholangiocyte markers. (TIF) [file pone.0200416.s001.tif]

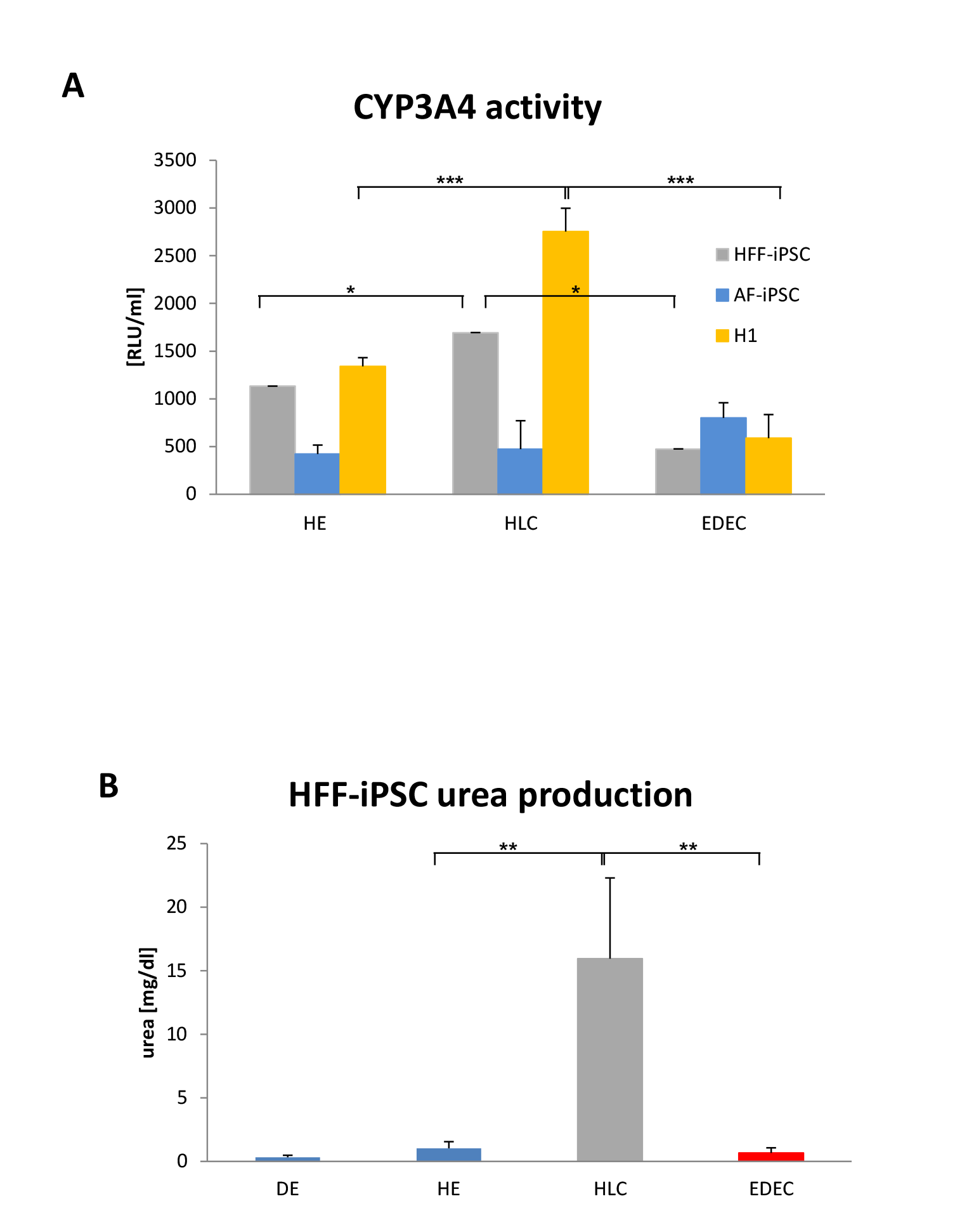

Supplement: S2 Fig — hPSC were differentiated into HLCs and EDECs. (A) CYP3A4 activity assay (B) QuantiChrome Urea Assay. (TIF) [file pone.0200416.s002.tif]

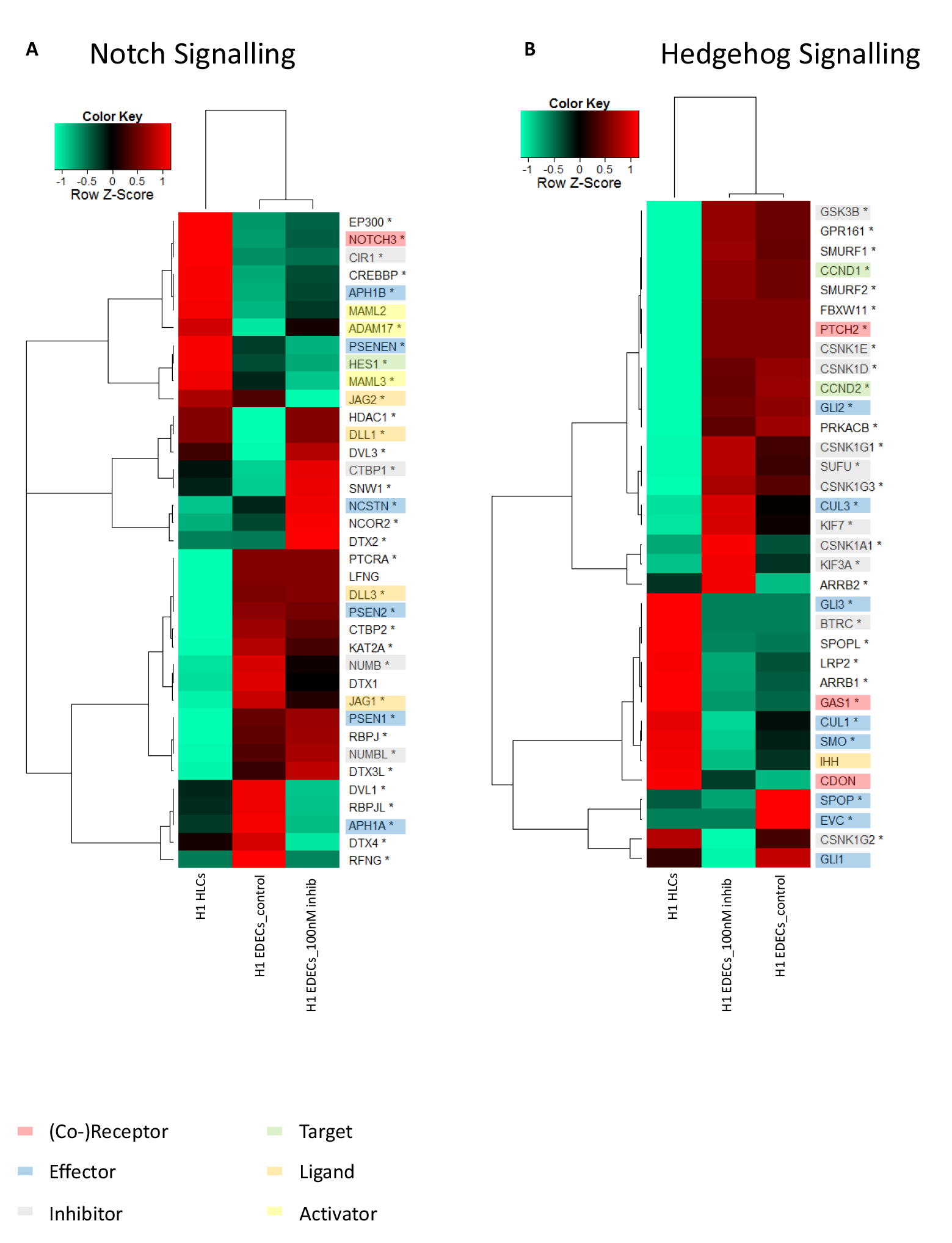

Supplement: S3 Fig — Global expression patterns of genes involved in Notch (A) and Hedgehog (B) signalling were analysed in HLCs and EDECs with and without Notch inhibitor. Genes were colour-coded according to their function. Asterisks mark the genes that are expressed above threshold in at least the EDEC sample or the EDEC sample with inhibitor. (TIF) [file pone.0200416.s003.tif]

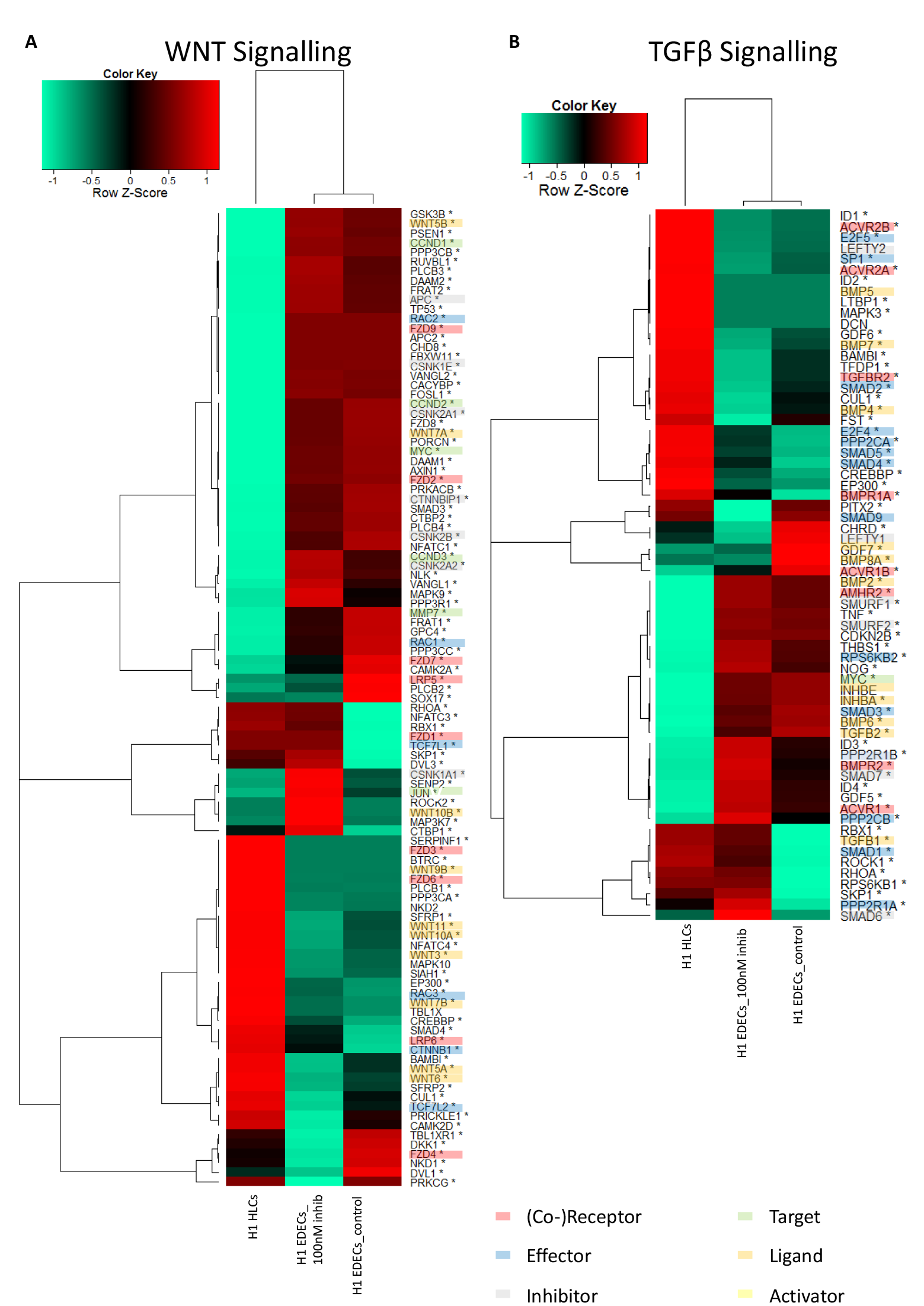

Supplement: S4 Fig — Global expression patterns of genes involved in WNT (A) and TGFβ (B) signalling were analysed in HLCs and EDECs with and without Notch inhibitor. Genes were colour-coded according to their function. Asterisks mark the genes that are expressed above threshold in at least the EDEC sample or the EDEC sample with inhibitor. (TIF) [file pone.0200416.s004.tif]
