# Supplementary material for: Cell fate decisions of human iPSC-derived bipotential hepatoblasts depend on cell density
Source: PLoS One. 2018 Jul 10;13(7):e0200416. doi: 10.1371/journal.pone.0200416 (PMC6039024; doi:10.1371/journal.pone.0200416)
Supplement: S1 Table — (DOCX) [file pone.0200416.s005.docx]

| Name | Function | Concentrations used | Company |
| --- | --- | --- | --- |
| PKF 118-310 | Inhibitor of WNT signalling | 5 µM / 0.1 µM | Calbiochem |
| Chir99021 | Activator of WNT signalling | 5 µM / 1.25 µM | Tocris |
| Cyclopamine-KAAD | Inhibitor of Hedgehog signalling | 0.5 µM / 0.125 µM | Calbiochem |
| Purmorphamine | Activator of Hedgehog signalling | 2 µM / 0.5 µM | Tocris |
| A-83-01 | Inhibitor of TGFβ signalling | 5 µM / 1.25 µM | Tocris |
| SB431242 | Inhibitor of TGFβ signalling | 10 µM / 2.5 µM | Sigma |
| Compound E | Inhibitor of Notch signalling | 0.1 µM / 0.5 µM /1 µM | Calbiochem |
| y-Secretase Inhibitor I | Inhibitor of Notch signalling | 50 nM / 100 nM | Calbiochem |

**Supplementary Table S1: small molecules**
