# Supplementary material for: Cell fate decisions of human iPSC-derived bipotential hepatoblasts depend on cell density
Source: PLoS One. 2018 Jul 10;13(7):e0200416. doi: 10.1371/journal.pone.0200416 (PMC6039024; doi:10.1371/journal.pone.0200416)
Supplement: S2 Table — (DOCX) [file pone.0200416.s006.docx]

**Supplementary Table S2: Antibodies**

| Antibody | Company | Order Number | dilution |
| --- | --- | --- | --- |
| Rabbit anti AFP | Sigma | HPA023600 | 1:200 |
| Mouse anti Albumin | Sigma | A6684-.2ml | 1:500 |
| Mouse anti CDX2 | Santa Cruz | Sc-166830 | 1:200 |
| Mouse anti CFTR | Abcam | ab2784 | 1:500 |
| Rabbit anti CK19 | Novus Biologicals | NB100-687 | 1:100 |
| Rabbit anti E-Cadherin | CST | 3195 | 1:200 |
| Guinea pig anti GFAP | SySy | 173004 | 1:500 |
| Rabbit anti HNF4A | Abcam | 92378 | 1:250 |
| Goat anti OPN | R&D | AF1433 | 1:150 |
| Rabbit anti SLC10A2 | Sigma | HPA004795-100µl | 1:200 |
| Rabbit anti SOX9 | Millipore | AB5535 | 1:400 |
