# Supplementary material for: Cell fate decisions of human iPSC-derived bipotential hepatoblasts depend on cell density
Source: PLoS One. 2018 Jul 10;13(7):e0200416. doi: 10.1371/journal.pone.0200416 (PMC6039024; doi:10.1371/journal.pone.0200416)
Supplement: S3 Table — (DOCX) [file pone.0200416.s007.docx]

**Supplementary Table S3: Primer Sequences**

| Gene | Sequence 5'-3' | Product size [bp] |
| --- | --- | --- |
| sAFP | AGCAGCTTGGTGGTGGATGA | 88 |
| asAFP | CCTGAGCTTGGCACAGATCCT |  |
| sALB | AGCTGTTATGGATGATTTCGCAG | 77 |
| asALB | CCTCGGCAAAGCAGGTCTC |  |
| sCEBPA | GGTGGACAAGAACAGCAACGA | 136 |
| asCEBPA | GTCATTGTCACTGGTCAGCTC |  |
| sCK18 | GAGGTTGGAGCTGCTGAGAC | 99 |
| asCK18 | CAAGCTGGCCTTCAGATTTC |  |
| sCK19 | CCGCGACTACAGCCACTACT | 100 |
| asCK19 | ATTGTCGATCTGCAGGACAATC |  |
| sCYP3A4 | GTGACTTTGCCCATTGTTTAGAAAG | 79 |
| asCYP3A4 | CAGGCGTGAGCCACTGTG |  |
| sFOXA2 | TTCAGGCCCGGCTAACTCTG | 97 |
| asFOXA2 | CCTTGCGTCTCTGCAACACC |  |
| sHNF4A | GTGCGGAAGAACCACATGTACTC | 102 |
| asHNF4A | GAAGCATTTCTTGAGCCTGCAGTA |  |
| sOPN | GTGGCCACATGGCTAAACCCT | 74 |
| asOPN | GACTTACTTGGAAGGGTCTGTGG |  |
| sPROX1 | AGGGCTCTGAACATGCACTAC | 126 |
| asPROX1 | AGGATCAACATCTTTGCCTGC |  |
| sRPS16 | GCTATCCGTCAGTCCATCTCCAA | 73 |
| asRPS16 | CCTTCTTGGAAGCCTCATCCAC |  |
| sSOX9 | GACTTCTGAACGAGAGCGAGA | 125 |
| asSOX9 | CCCGTTCTTCACCGACTTCCT |  |
