# Supplementary material for: Cell fate decisions of human iPSC-derived bipotential hepatoblasts depend on cell density
Source: PLoS One. 2018 Jul 10;13(7):e0200416. doi: 10.1371/journal.pone.0200416 (PMC6039024; doi:10.1371/journal.pone.0200416)
Supplement: S6 Table — (DOCX) [file pone.0200416.s010.docx]

**Supplementary Table S6: Selected GO categories up- and down regulated in EDECs versus HLCs**

| **GO-Categories up-regulated in EDECs vs. HLCs** |  |  |  |  |
| --- | --- | --- | --- | --- |
| Term | Count | % | PValue | selected genes |
| regulation of apoptosis | 219 | 6.96 | 1.02E-12 | MEF2C, HRAS, PTGS2, STAT5A, GDF5, STAT5B, ADORA1, BAK1, MAP3K5, BDNF, HTRA2 |
| cell motion | 137 | 4.35 | 5.24E-10 | PVR, EDN3, PDGFB, PTGS2, PLXNA2, S100A9, L1CAM, TNFSF12, TGFB2, NRCAM, BDNF, CD44 |
| apoptosis | 158 | 5.02 | 3.31E-08 | MEF2C, MEF2A, HRAS, TSPO, FASTKD1, MRPL41, GRIK2, TNFSF12, PMAIP1, FOXO3, MRPS30 |
| cytoskeleton organization | 122 | 3.88 | 3.45E-08 | CEP72, BMP10, HRAS, PDGFB, PDLIM7, S100A9, PDLIM3, TTN, TTL, ANK1, TUBG1 |
| intracellular signaling cascade | 290 | 9.22 | 6.66E-08 | EDN3, RP1, HRAS, ADCY2, STAT5A, EDN1, STAT5B, ADORA1, MAP3K5, S1PR1, STAC |
| regulation of cell migration | 59 | 1.87 | 7.70E-08 | RTN4, BMP10, EDN3, PDGFB, PDGFA, F2RL1, EDN1, ADORA1, TGFB2, S1PR1, SERPINE2 |
| induction of apoptosis | 94 | 2.99 | 1.37E-07 | PREX1, ZMAT3, TLR2, TNFSF12, FOXO3, PMAIP1, DAXX, ADORA1, PRDX1, ARHGAP4, BAK1 |
| regulation of cell proliferation | 191 | 6.07 | 5.27E-07 | EDN3, RARRES3, BMP10, HRAS, S100A6, NOG, RARRES1, CNOT8, PDGFB, PTGS2, PDGFA |
| positive regulation of developmental process | 82 | 2.61 | 7.78E-07 | ZNF488, BMP10, MSR1, PDLIM7, STAT5A, GDF5, STAT5B, NFKB1, TNFSF12, FOXO3, GLI2 |
| cell adhesion | 170 | 5.4 | 2.29E-06 | MPZL3, PVR, CADM3, CADM4, MPZL2, CASK, L1CAM, EDIL3, MEGF10, NRCAM, CDH22 |
| cell proliferation | 109 | 3.46 | 5.17E-05 | TUSC2, STIL, BMP10, TSPO, PDGFB, PDGFA, OSMR, IL15, TGFB2, ISG20, BAK1 |
| regulation of cell development | 57 | 1.81 | 2.51E-04 | RTN4, ZNF488, NOG, LZTS1, EDN1, LRRC4C, GLI2, TTL, TGFB2, NRCAM, METRN |
| response to oxidative stress | 47 | 1.49 | 4.59E-04 | MICB, PXDN, GEDEC, PTGS2, PML, TLR4, RRM2B, BCL2L1, CCL5, GCLM, PRDX1 |
| response to hormone stimulus | 89 | 2.83 | 7.59E-04 | ADCY2, PTGS2, PDGFB, PDGFA, STAT5A, LEPR, STAT5B, RHOQ, CUZD1, RPE65, AQP1 |
| positive regulation of cell development | 22 | 0.7 | 0.006 | ZNF488, LIMK1, TNFRSF12A, PLXNB2, MAP1B, DLL3, SMAD3, CDH4, NUMBL, EPHB2, TGFB2 |
| regulation of cell cycle | 75 | 2.38 | 0.012 | MAD1L1, DYNC1LI1, EDN3, PTGS2, STAT5A, EDN1, STAT5B, MOV10L1, TGFB2, BAK1, SBDS |
| cell-cell adhesion | 64 | 2.03 | 0.012 | PVR, PCDHA7, CADM3, MPZL2, CLSTN1, L1CAM, NRCAM, CDH22, PCDH1, CD44 |
| cell-cell signaling | 126 | 4 | 0.015 | TUSC2, SYT1, EDN3, KCNC4, GABRB3, PDGFA, GRIK2, IL18, LTBP4, GDF5, S100A9 |
| cell cycle arrest | 28 | 0.89 | 0.016 | PML, SESN2, MLF1, TGFB2, CDKN2A, MACF1, CDKN2B, PCBP4, CDC123, THBS1, MYC |
| regulation of transcription factor activity | 27 | 0.86 | 0.028 | TNF, MTDH, CYTL1, TLR2, NFKBIA, TLR4, CDKN2A, SMARCB1, GTF2A2, TICAM1, PYCARD |
| **GO-Categories down-regulated in EDECs vs. HLCs** |  |  |  |  |
| Term | Count | % | PValue | selected genes |
| regulation of transcription | 545 | 17.87 | 2.01E-08 | ZNF57, RORA, ZNF253, CTNNB1, MAGED1, APP, CDCA7, MED28, PHTF2, ZNF397, ZNF396 |
| cell cycle phase | 114 | 3.74 | 1.20E-07 | PRC1, DBF4, KNTC1, TTK, PTTG1, FOXO4, APP, RAD21, OIP5, CDCA2, CCNA2 |
| tube development | 67 | 2.2 | 1.66E-06 | GNA13, NRP1, PGF, PDGFA, LMO4, TP63, GJA5, GLI3, LGR4, CTNNB1 |
| enzyme linked receptor protein signaling pathway | 94 | 3.08 | 1.87E-06 | MPZL1, NRTN, NRP1, IL6ST, PDGFA, LTBP3, GDF6, SHOC2, FOXO1, FSTL1, FOXO4 |
| cell cycle process | 137 | 4.49 | 1.31E-05 | CDC25C, CDKN3, SMC2, CENPJ, SMC4, CDC25B, CCNB1, MPHOSPH9, CCNB2, CIT, BARD1 |
| chromosome organization | 119 | 3.9 | 2.96E-05 | HIST2H2AA3, HMGN2, RBM4, MORF4L2, PTTG1, CBX5, TLK1, ACIN1, CDCA5, H1F0, SATB1 |
| response to extracellular stimulus | 62 | 2.03 | 5.75E-05 | ARSB, CYP24A1, PPARA, KYNU, IL6ST, PDGFA, IGFBP7, PPARG, AQP3, FOS, CCNE1 |
| response to endogenous stimulus | 100 | 3.28 | 1.05E-04 | CGA, ADCY7, PDGFA, PGF, RBM4, FOXO1, FOXO4, CTNNB1, CTTNBP2, APOA2, EIF4EBP2 |
| cellular response to stress | 132 | 4.33 | 1.28E-04 | XRCC4, ZAK, RBM4, MORF4L2, PTTG1, FOXO4, APOA4, NONO, MUTYH, RAD21, MDFIC |
| cell proliferation | 104 | 3.41 | 3.04E-04 | LMO1, ZAK, PDGFA, PGF, ENPEP, CLK1, CTNNB1, DAB2, APOA1, GAB1, TGFBI |
| BMP signaling pathway | 18 | 0.59 | 5.85E-04 | BMP4, USP9Y, SMAD6, USP9X, GDF6, SMAD5, BMPR2, SMAD4, FSTL1, GREM2, MSX2 |
| TGFβ receptor signaling pathway | 22 | 0.72 | 6.65E-04 | FUT8, PDGFA, USP9Y, LTBP3, KLF10, CREB1, TGFBR1, SMAD6, USP9X, SMAD5, COL3A1 |
| cell adhesion | 149 | 4.89 | 0.003 | ITGB3BP, LYPD3, CADM1, NELL2, POSTN, PNN, CTNNB1, APOA4, CD47, APP, DAB1 |
| DNA metabolic process | 109 | 3.57 | 0.008 | XRCC4, DBF4, RBM4, MORF4L2, PTTG1, MCM10, NONO, MUTYH, RAD21, PMS1, ESCO1 |
| Wnt receptor signaling pathway | 33 | 1.08 | 0.026 | WNT5A, NKD2, PPM1A, CTNND1, TCF7L2, CALCOCO1, CPZ, CTNNB1, WNT3, WISP1, SOSTDC1 |
| fat cell differentiation | 16 | 0.52 | 0.03 | CEBPA, ALDH6A1, SOCS1, PPARG, RUNX1T1, TTC8, TCF7L2, SIRT1, PPARGC1A, PEX11A, LAMA4 |
| steroid hormone receptor signaling pathway | 17 | 0.56 | 0.032 | GRIP1, RBM4, MED12, FHL2, ARID1A, MED13, PPARGC1A, CALCOCO1, BRCA1, CTNNB1, NRIP1 |
| posttranscriptional regulation of gene expression | 48 | 1.57 | 0.033 | EIF4E3, CPEB2, RBM3, EIF5, PRKDC, PAIP2B, ZFP36L1, ASGR2, ZFP36L2, TNFRSF1B, APP |
| cell motion | 98 | 3.21 | 0.035 | GNA13, CTHRC1, NRTN, LYPD3, HMGCR, ENPEP, MYLIP, PRKG1, CTTNBP2, APP, APOB |
| regulation of cell development | 46 | 1.51 | 0.045 | IRX3, XRCC4, NBN, NRP1, PPARG, SOX5, KIT, TTC3, ROBO1, SEMA3F, S1PR5 |
