# Supplementary material for: Cell fate decisions of human iPSC-derived bipotential hepatoblasts depend on cell density
Source: PLoS One. 2018 Jul 10;13(7):e0200416. doi: 10.1371/journal.pone.0200416 (PMC6039024; doi:10.1371/journal.pone.0200416)
Supplement: S9 Table — (DOCX) [file pone.0200416.s013.docx]

**Supplementary Table S9: Selected GO categories up- and down regulated in EDECs with y-secretase inhibitor versus untreated EDECs**

| **GO-categories up-regulated in EDECs with y-secretase inhibitor vs. EDECs** |  |  |  |  |
| --- | --- | --- | --- | --- |
| Term | Count | % | PValue | Genes |
| regulation of chromosome organization | 8 | 0.99 | 3.39E-04 | PIF1, TNKS, ESPL1, TLK1, PPARGC1A, POT1, TERF1, TNKS2 |
| cellular macromolecule localization | 35 | 4.33 | 7.64E-04 | GRPEL2, OXA1L, AP1G1, NAPB, CTNNB1, GCKR, TRAK2, CEP57, EXOC4, CHM, PEX14 |
| intracellular transport | 49 | 6.06 | 9.68E-04 | GRPEL2, ATL2, AP1G1, WASF2, TAPBP, HOOK1, TRAK2, DDX25, VPS4B, DNAJC6, RANBP1 |
| regulation of telomere maintenance | 5 | 0.62 | 0.002 | PIF1, TNKS, POT1, TERF1, TNKS2 |
| DNA replication | 18 | 2.23 | 0.007 | GINS1, CLSPN, POLK, POLI, MRE11A, GINS4, NFIX, RRM2B, SIRT1, POT1, CDC25A |
| cell cycle | 51 | 6.31 | 0.009 | CCNT2, GAS2L3, CLSPN, ZAK, E2F8, TTN, SART1, CTNNB1, CDCA2, VPS4B, RANBP1 |
| regulation of DNA metabolic process | 12 | 1.49 | 0.016 | FAM175A, PIF1, MRE11A, PDGFRA, TP53, RNF168, TNKS, CD40, GLI2, POT1, TERF1, TNKS2 |
| vesicle-mediated transport | 38 | 4.7 | 0.025 | CCL3, MSR1, ATL2, PDLIM7, AP1G1, GULP1, WASF2, NAPB, SCARF1, TAPBP, CTNNB1 |
| positive regulation of developmental process | 21 | 2.6 | 0.032 | B4GALT1, MSR1, PDLIM7, IL6R, SOCS5, GLI2, VASH2, NTN1, SHH, SART1, CTNNB1 |
| lipopolysaccharide-mediated signaling pathway | 3 | 0.37 | 0.088 | IRAK2, MAPK14, STAT1 |
| **GO-Categories down-regulated in EDECs with y-secretase inhibitor vs. EDECs** |  |  |  |  |
| Term | Count | % | PValue | Genes |
| cell-cell signaling | 65 | 6.47 | 5.55E-07 | NAMPT, INSL3, CGA, HNF1B, NRP1, SYT4, FAM3B, LTBP4, SLC6A4, EDN1, FASLG |
| tube development | 33 | 3.29 | 7.42E-07 | WNT5A, RBP4, NRP1, EDN1, GDNF, GLI1, SEMA5A, WNT4, BDNF, GPC3, HAND1 |
| embryonic organ development | 28 | 2.79 | 1.21E-06 | RBP4, EDN1, COL2A1, TCF7L2, ARNT, GLI1, GATA2, OSR2, CHRNA9, HAND1, HOXA7 |
| cell adhesion | 69 | 6.87 | 6.78E-06 | NRP2, CADM3, NRP1, CLSTN3, BCAN, CUZD1, POSTN, FER, CDH22, ARHGAP6, VNN1 |
| regulation of cell proliferation | 70 | 6.97 | 1.52E-04 | NAMPT, XRCC4, NRP1, PTGS2, EDN1, MITF, MMP7, SCGB1A1, FLT3LG, GLI1, EDNRB |
| sterol transport | 10 | 1 | 1.64E-04 | APOA2, APOB, MSR1, CD36, APOA1, APOC1, APOC2, CFTR, CETP, AKR1C1 |
| positive regulation of developmental process | 31 | 3.09 | 4.86E-04 | XRCC4, MSR1, FST, KITLG, ARNT, GATA2, APOB, BDNF, VNN1, NOS3, ROBO2 |
| anion transport | 19 | 1.89 | 0.001 | GABRA2, FXYD3, GABRA6, ENPP3, GLRA2, SLC34A1, CFTR, SLC26A1, ANKH, SLC34A2, BEST1 |
| positive regulation of cell differentiation | 26 | 2.59 | 0.001 | XRCC4, MSR1, KITLG, ARNT, APOB, BDNF, VNN1, ROBO2, INPP5D, RUNX2, LPL |
| Wnt receptor signaling pathway | 16 | 1.59 | 0.008 | WNT5A, RARG, MITF, CXXC4, TLE2, TCF7L2, CALCOCO1, FZD10, WNT7B, WNT4, WNT3 |
| lipid transport | 15 | 1.49 | 0.034 | RBP4, SLC27A1, MSR1, ATP10B, APOC1, APOC2, CFTR, STARD3, APOA2, APOB, CD36 |
